# Supplementary material for: Iron-dependent histone 3 lysine 9 demethylation controls B cell proliferation and humoral immune responses
Source: Nat Commun. 2019 Jul 3;10:2935. doi: 10.1038/s41467-019-11002-5 (PMC6610088; doi:10.1038/s41467-019-11002-5)
Supplement: Supplementary file 1 — Supplementary information [file 41467_2019_11002_MOESM1_ESM.pdf]

Supplementary Information

Iron-dependent histone 3 lysine 9 demethylation controls B cell proliferation and humoral immune responses

Jiang, et al.

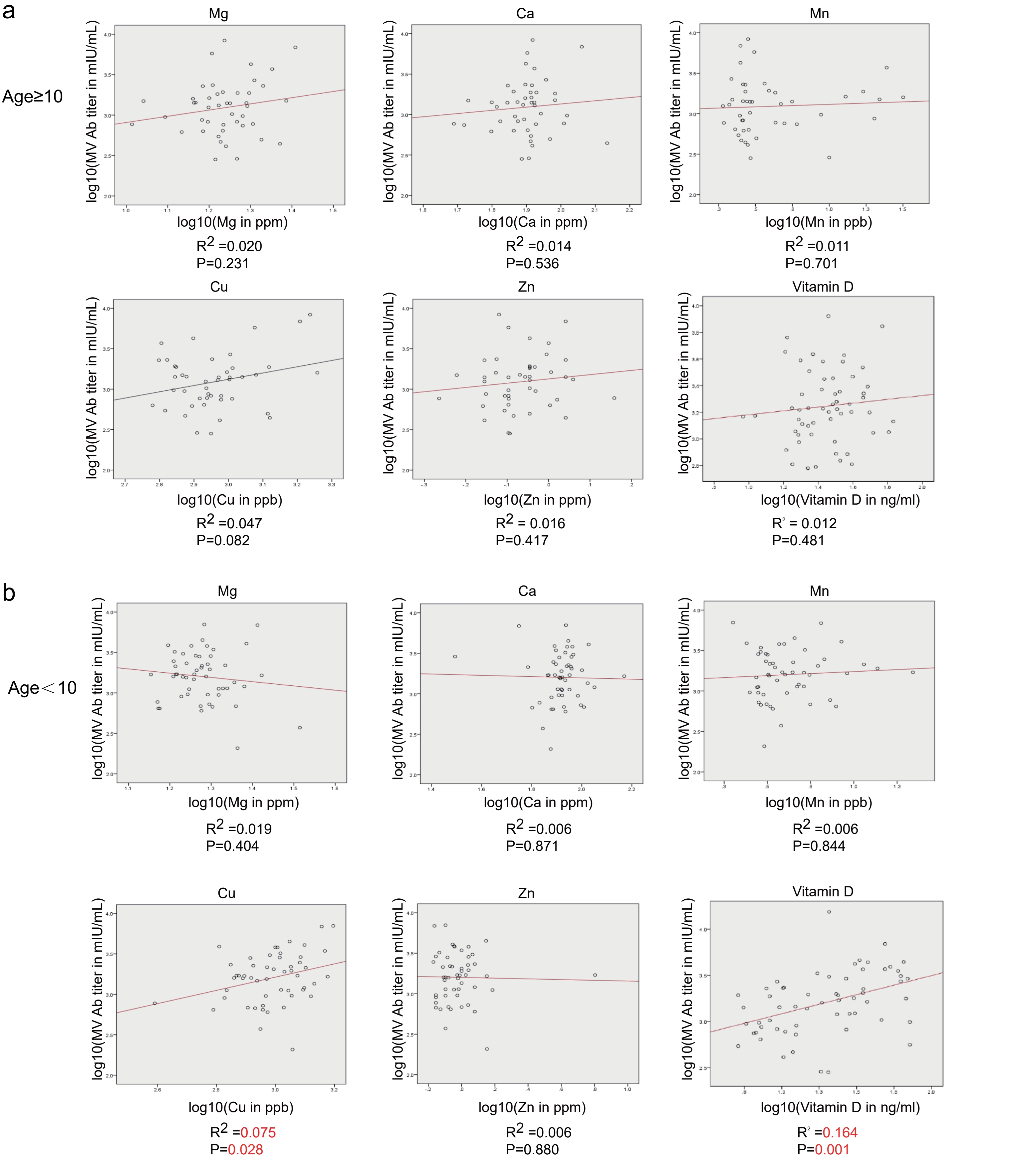

Supplementary Figure 1. Involvement of other trace elements and vitamin D in humoral immune response. (a and b) A linear regression model was used to analyze the relationship between trace elements (Magnesium, calcium, manganese, copper, zinc) and MV-specific antibody titers, between vitamin D and MV-specific antibody titers in age≥10 human subjects(a) and age＜10 subjects. (b) Trace elements were checked by ICP-MS, and vitamin D was checked by ELISA. Pearson’s correlation test was used to determine the correlation coefficient r. P < 0.05 was considered significant (*P < 0.05, **P < 0.01).

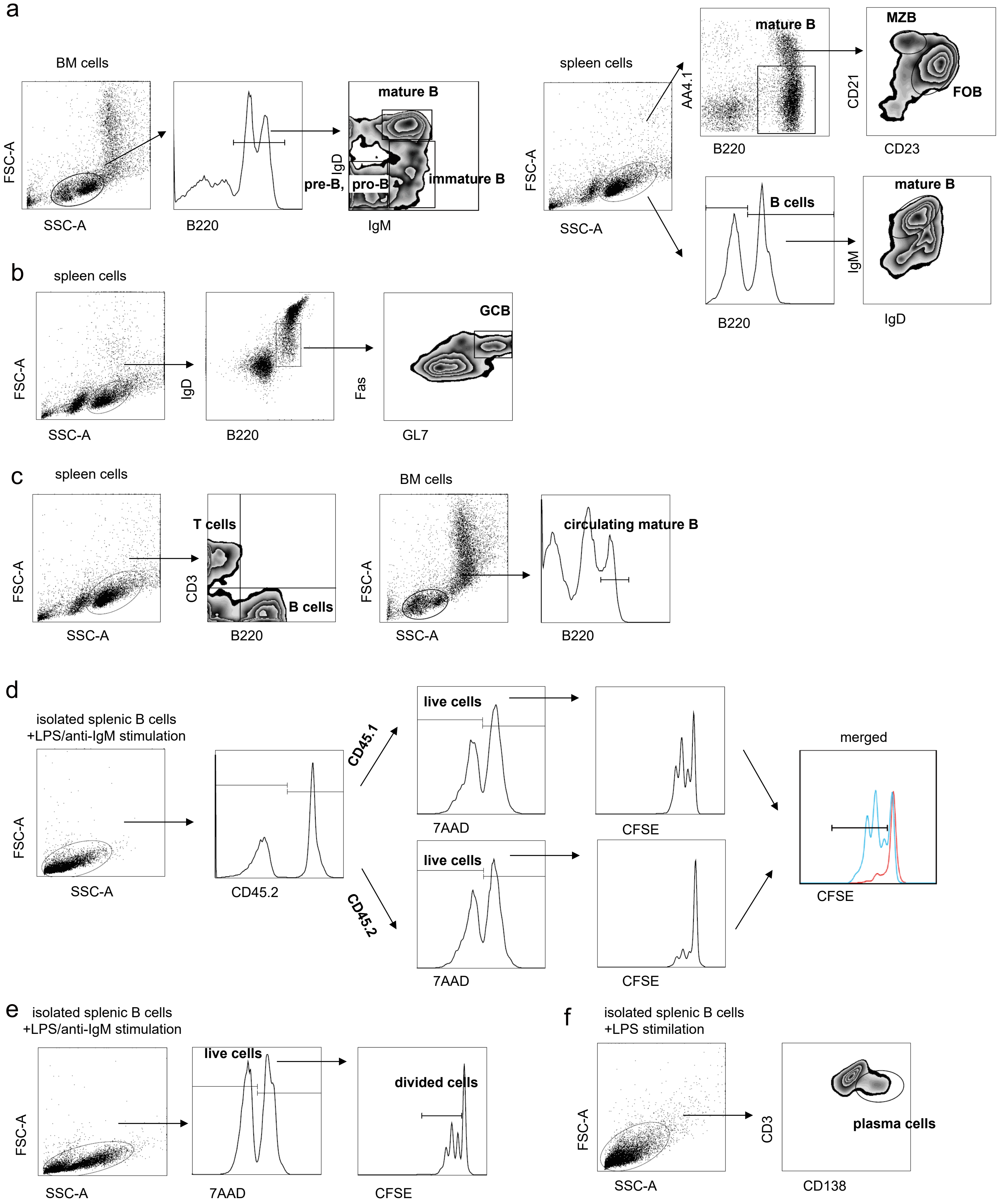

Supplementary Figure 2. Gating strategies used for cell sorting. (a) Gating strategy of mature B cells (B220+IgM+IgDhi) in bone marrw, MZB (B220+AA4.1-CD21hiCD23int) and FOB(B220+AA4.1-CD21intCDhi) differentiation in spleen, as presented on Fig. 2a,b. (b) Gating strategy of germinal center B cells (B220+IgDlowFas+GL7+) from wildtype and iron-deficient mice immunized with antigens. as presented on Fig. 2f, g. (c) Gating strategy of B cells and circulating B cells in bone marrow, as presented on Fig. 3a and supplementary Fig. 4. (d) Gating strategy to assess B cell proliferation (divided cells) of isolated B cells from co-transfer mice,as presented on Fig. 3e-h. (e) Gating strategy to assess B cell proliferation of isolated B cells from wildtype C57BL/6 mice. The same strategy was used for the in vitro proliferation assay presented on Fig. 4c, 5c-d, 7a and 8d. (f) Gating strategy of plasma cells for in vitro cultures presented on Fig. 4e.

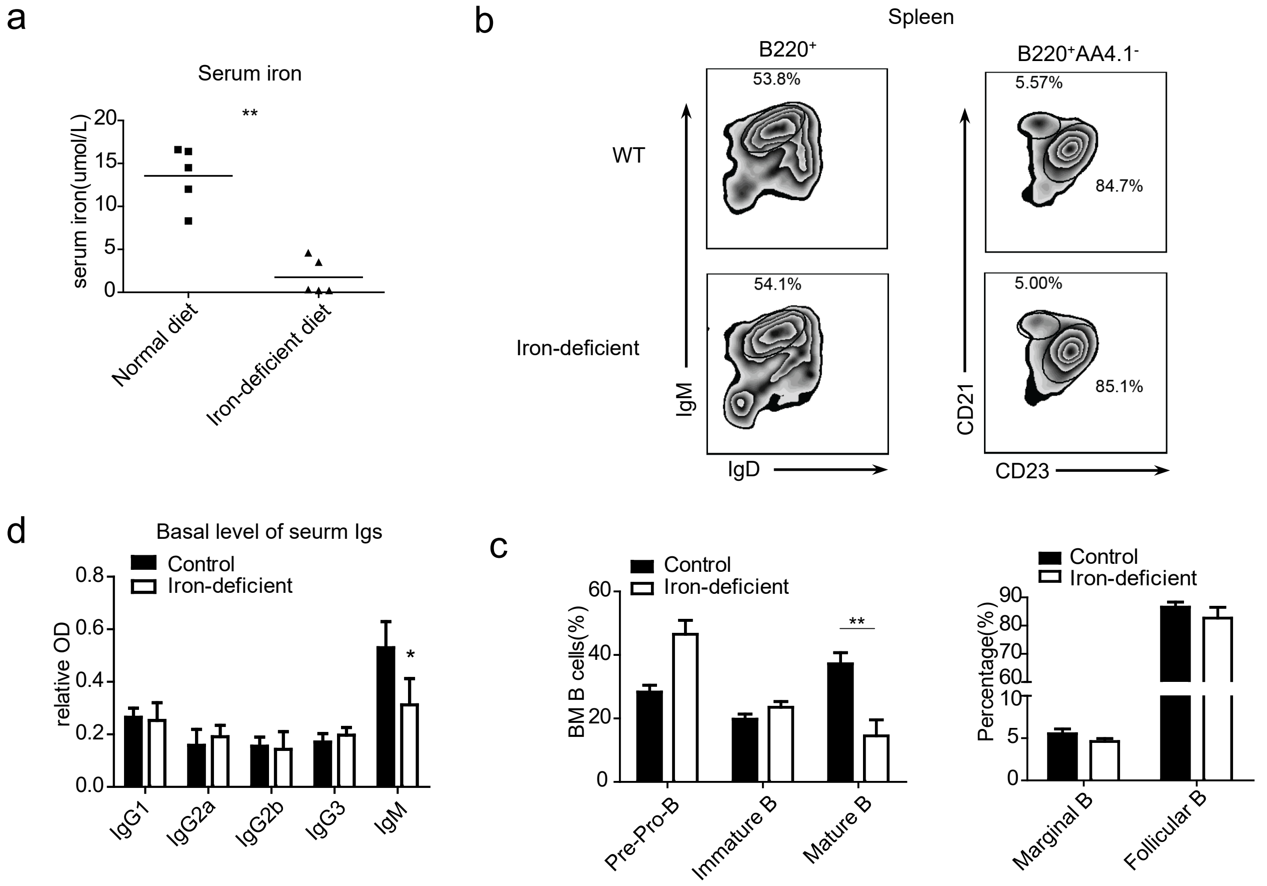

Supplementary Figure 3. Iron-deficient mouse model. (a) Serum iron levels in the peripheral blood serum of mice that were fed a control diet or an iron-deficient diet for 5 weeks. (b) Flow cytometry of splenocytes from control and iron-deficient mice. The splenocytes were stained with anti-B220 to identify B cells and further stained with anti-IgM and anti-IgD to assess B cell maturation. Evaluation of MZB cells and FOB cells was followed by staining with anti-AA4.1, anti-CD21 and anti-CD23 antibodies. (c) Statistics for the percentage of B cell subsets in the two groups (mean ± SEM of three mice per group). (d) Results of ELISA to test basal levels of different serum Ig isotypes (mean ± SEM of four mice per group). *P < 0.05, **P < 0.01, Student's t test.

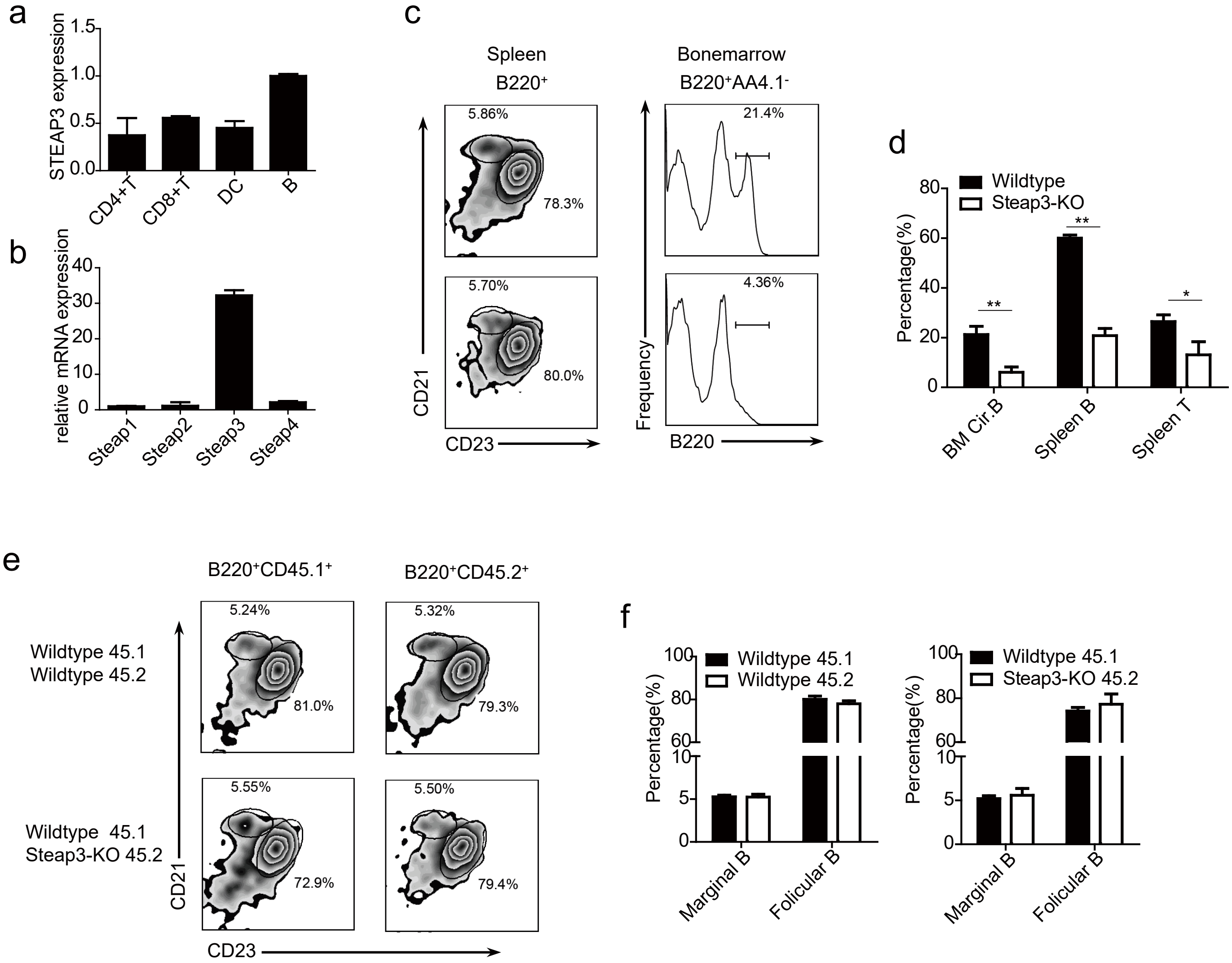

Supplementary Figure 4. High STEAP3 expression in B cells and B cell differentiation in Steap3-KO mice.
(a) STEAP3 expression in primary CD4+ T cells, CD8+ T cells, dendritic cells and B cells purified from the spleens of wild-type C57BL/6J mice (males, 8 weeks old). The expression levels were normalized to GAPDH expression, and the expression level of STEAP3 in primary B cells was set as 1. (b) Relative mRNA expression levels of STEAP1, STEAP2, STEAP3 and STEAP4 in splenic B cells. (c) Evaluation of MZB cells (B220+AA4.1-CD21hiCD23int) and FOB cells (B220+AA4.1-CD21intCD23hi) in the spleen (c, left). Identification of mature circulating B cells (B220hi) in bone marrow (c, right). (d) Statistics for the percentage of mature circulating B cells in bone marrow and the percentage of splenic B and T cells in the two groups (mean ± SEM of three mice per group). (e) Flow cytometry of splenocytes from recipient mice was conducted to compare CD45.1+ wild-type B cells with CD45.2+ wild-type or Steap3-KO B cells. The splenocytes were further stained with anti-CD21 and anti-CD23 antibodies to evaluate MZB cells and FOB cells. (f) Statistics for the percentage of CD45.1+ or CD45.2+ MZB cells and FOB cells in the spleens of different recipient mice (mean ± SEM of three mice per group). *P < 0.05, **P < 0.01, Student's t test.

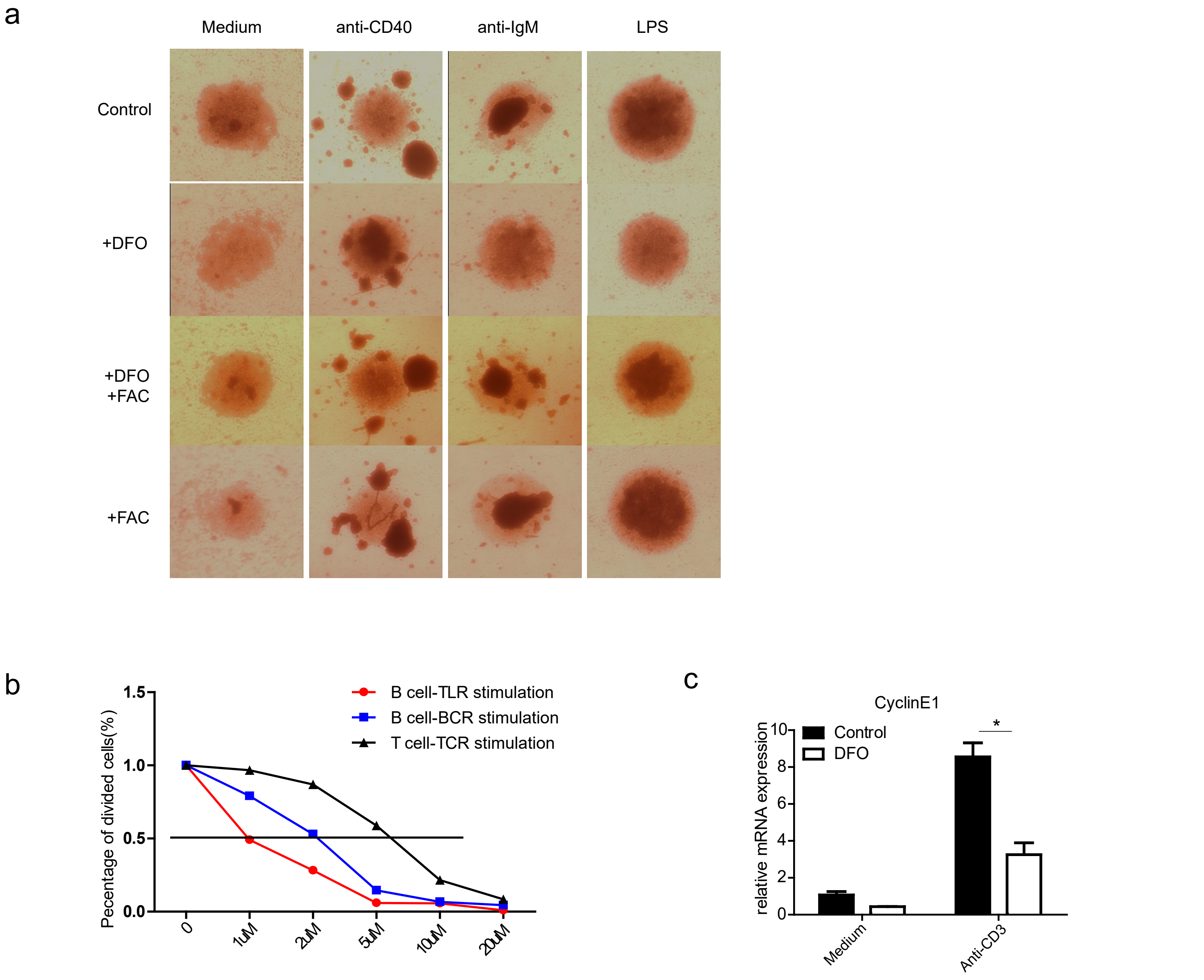

Supplementary Figure 5. Impaired B and T Cell proliferation under iron-deficient conditions in response to various stimulations. (a) Clonal expansion of B cells that were unstimulated or stimulated with anti-CD40 (1 μg/ml), anti-IgM (10 μg/ml) or LPS (2 μg/ml) for 48 h in the presence of DFO (20 μM), DFO and FAC (100 μM) or FAC (100 μM) alone in normal medium. Images were acquired using an Axio Observer.A1 microscope (Zeiss). (b) Splenic B cells were stimulated with anti-IgM (BCRs, 10 μg/ml) or LPS (TLRs, 2 μg/ml), and T cells were stimulated with anti-CD3 (TCRs, 1 μg/ml) in the presence of different concentrations of DFO. Proliferation was assessed by CFSE dilution 72 h later, and the percentage of divided cells in the absence of DFO was set as 1. (c) Cyclin E1 induction were measured by qRT-PCR in control and iron-deficient T cells (20uM DFO) that were unstimulated or stimulated with anti-CD3 (1 μg/ml). Data were representative of two independent experiments. *P < 0.05, **P < 0.01, Student's t test.

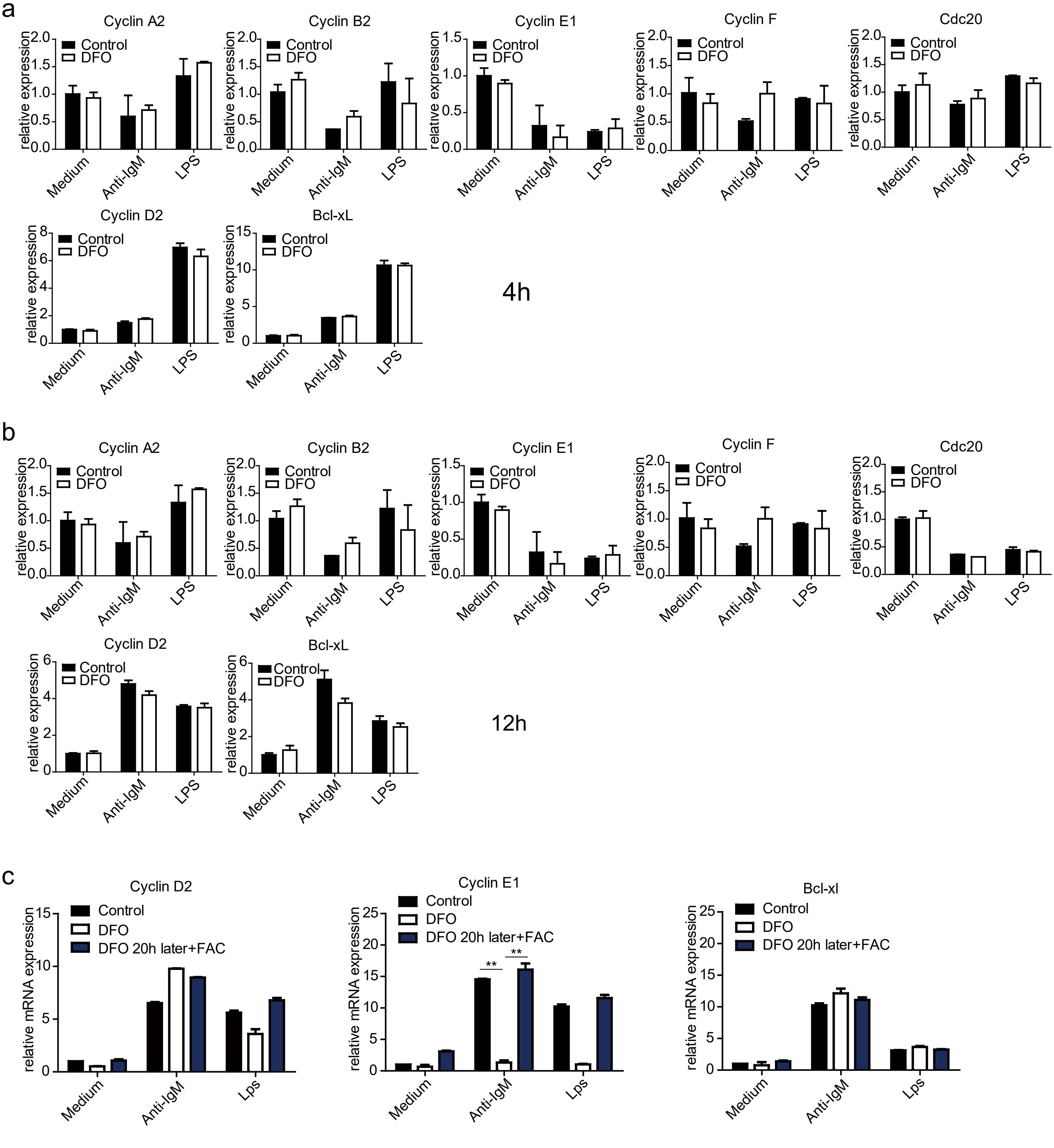

Supplementary Figure 6. Iron is not required for induction of cell cycle-related genes during early stage of B cell proliferation. (a and b) qRT-PCR analysis of the expression of a set of cell cycle-related genes and B cell survival-related Bcl-xL expression in control and iron-deficient B cells following BCR or TLR stimulation for 4 h (a) and 12 h (b). (c) Control and iron-deficient B cells were unstimulated or stimulated with anti-IgM (10 μg/ml) or LPS (2 μg/ml) for 48 h; in the third group, FAC was added to iron-deficient B cells 20 h after stimulation. The mRNA levels of cyclin D2, cyclin E1 and Bcl-xL were measured by qRT-PCR in the three groups. *P < 0.05, **P < 0.01, Student's t test.

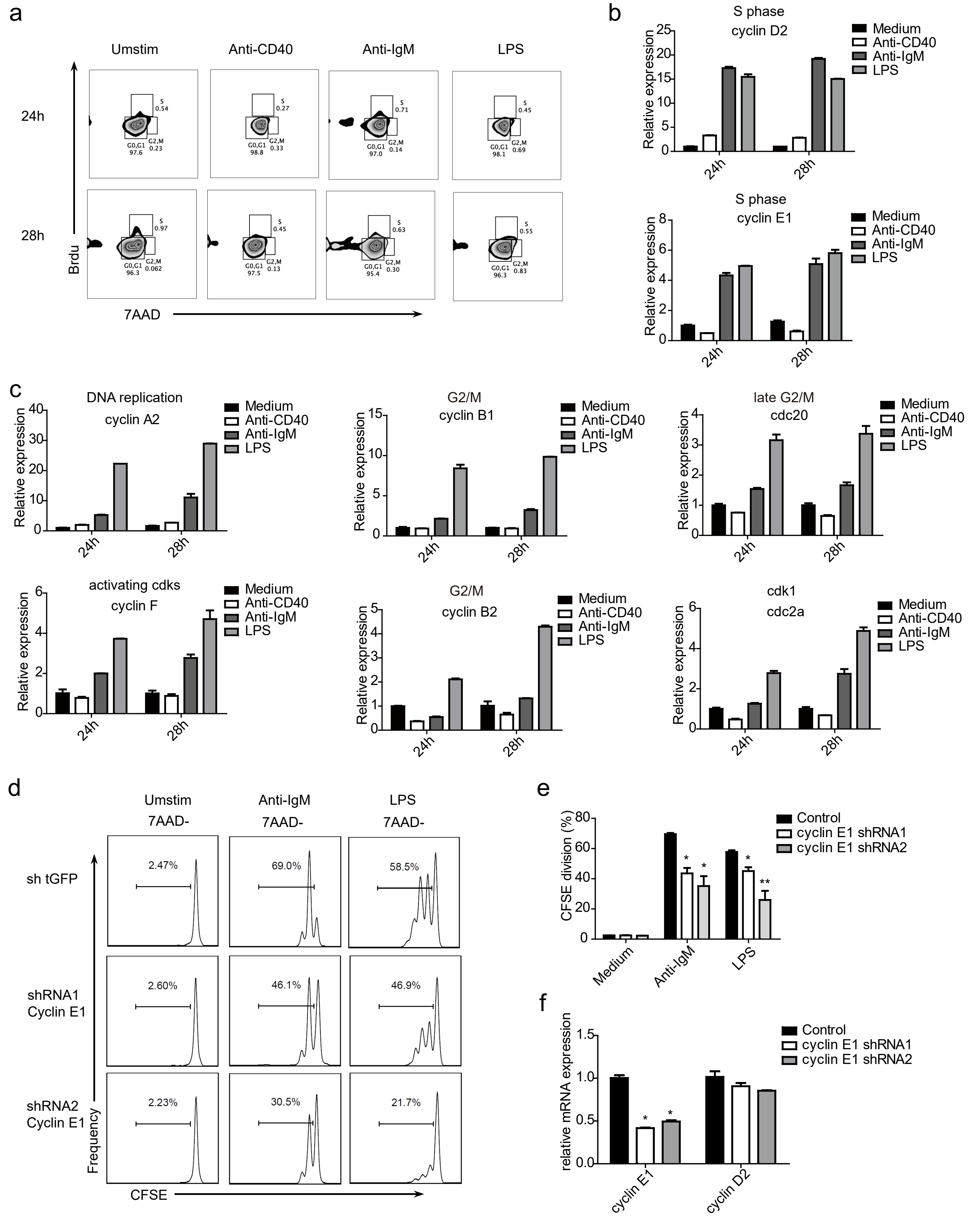

Supplementary Figure 7. Cyclin E1 and cyclin D2 induction occur prior to DNA replication in activated B cells. (a) Naive B cells were stimulated with anti-CD40 (1 μg/ml), anti-IgM (10 μg/ml) or LPS (2 μg/ml) for 24 or 28 h, cocultured with 10 μM BrdU for the last 45 min, stained with anti-BrdU and 7AAD, and analyzed by flow cytometry. (b and c) Induction of different cell cycle-related genes at 24 h and 28 h post stimulation. (d) CFSE-labeled splenic B cells from wild-type C57BL/6 mice were infected with cyclin E1 shRNA (two independent sequences) lentivirus, and cell proliferation was analyzed by flow cytometry following anti-IgM (10 μg/ml) or LPS (2 μg/ml) stimulation for 72 h. (e) Statistics for the percentage of B cells that underwent cell division after shRNA-mediated cyclin E1 knockdown. (f) Cyclin E1 and cyclinD2 mRNA expression in cyclin E1 shRNA lentivirus-infected B cells. *P < 0.05, **P < 0.01, Student's t test.

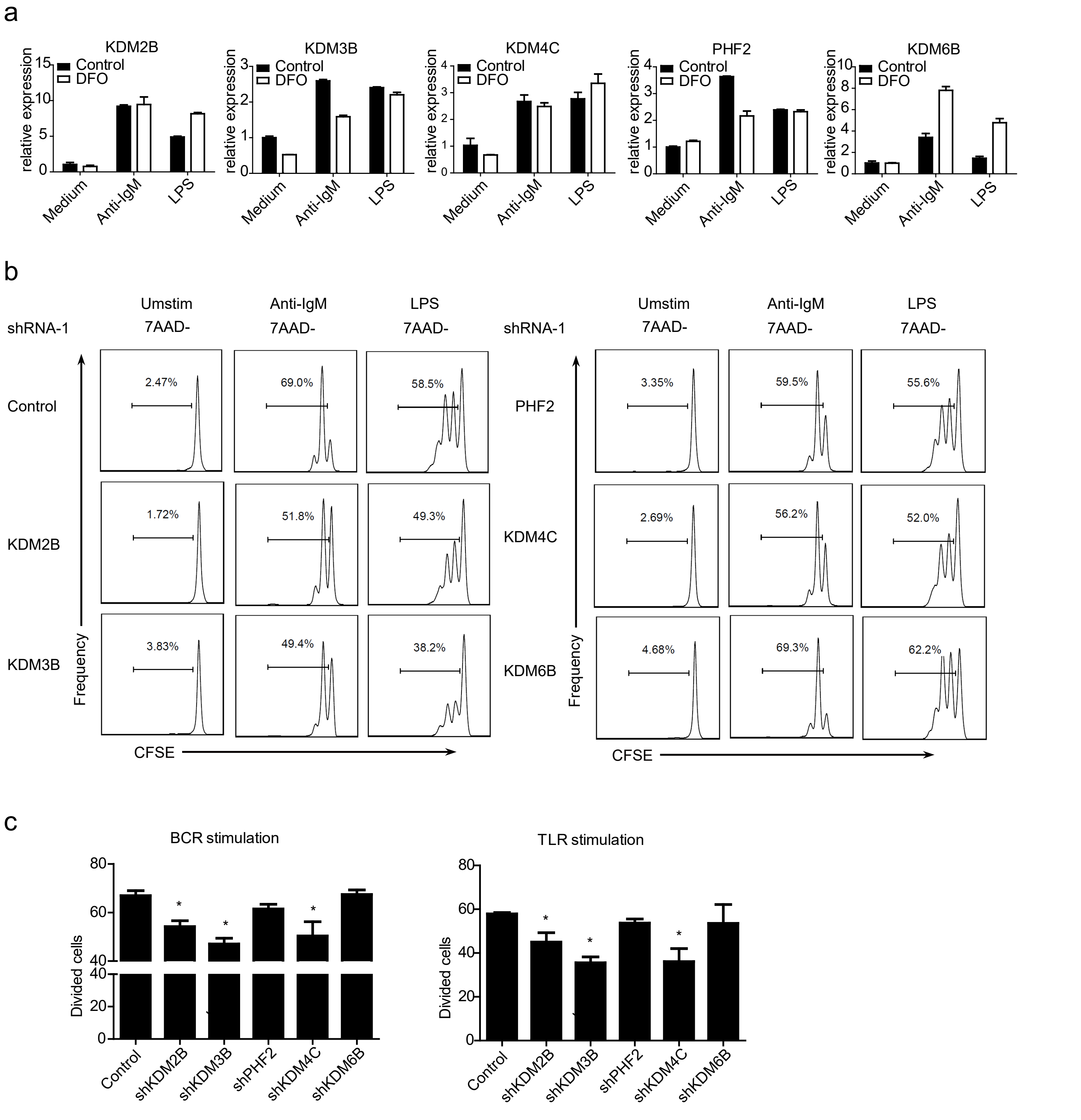

Supplementary Figure 8. KDM2B, KDM3B and KDM4C contribute to B cell proliferation to varying degrees. (a) qRT-PCR analysis of JmjC demethylase in control and iron-deficient B cells following BCR or LPS stimulation. The 5 JmjC demethylases induced after 48 h are shown. (b) CFSE-labeled splenic B cells from wild-type C57BL/6 mice were infected with shRNA lentiviruses targeting 5 different JmjC demethylases, and cell proliferation was analyzed by flow cytometry after stimulation with anti-IgM (10 μg/ml) or LPS (2 μg/ml) for 72 h. (c) Statistics for the percentage of B cells that underwent cell division under various conditions after shRNA-mediated knockdown of different JmjC demethylases. The data were representative of two independent experiments using two different shRNA target sequences. *P < 0.05, **P < 0.01, Student's t test.

Supplementary table 1. shRNA sequences targeting cyclin E1

Cyclin E1 shRNA1	Top strand	gatcc**GGAGGATCATGTTAAACAAAG**TTCAAGAGA**CTTTGTTTAACATGATCCTCC**TTTTTTg
	Bottom strand	aattcAAAAAA**GGAGGATCATGTTAAACAAAGT**TCTCTTGAA**CTTTGTTTAACATGATCCTCC**g
Cyclin E1 shRNA2	Top strand	gatcc**GCTTCTGCTTTGTATCATTTC**TTCAAGAGA**GAAATGATACAAAGCAGAAGC**TTTTTTg
	Bottom strand	aattcAAAAAA**GCTTCTGCTTTGTATCATTTC**TCTCTTGAA**GAAATGATACAAAGCAGAAGC**g
Control shRNA	Top strand	gatcc**GAAACCATGCAAAGTAAGGTT**TTCAAGAGA**AACCTTACTTTGCATGGTTTC**TTTTTTg
	Bottom strand	aattcAAAAAA**GAAACCATGCAAAGTAAGGTT**TCTCTTGAA**AACCTTACTTTGCATGGTTTC**g

Annealed top and bottom strands were inserted into pLVX-shRNA1 plasmid, EcoRI and BamHI were used as restriction enzymes.

Supplementary table 2. sequences of the RT-qPCR and ChIP-qPCR primers

**Real-time qPCR primer**	
Ccnf-F	TCAGGTTCTGTGGAGAAGGC
Ccnf-R	TTCTGTTGGGGACATCCTTG
Ccnb2-F	CTTGCAGAGCAGAGCATCAG
Ccnb2-R	TGAAACCAGTGCAGATGGAG
Ccne1-F	TCCACGCATGCTGAATTATC
Ccne1-R	CTCCAGAAAAAGGAAGGCAA
Ccnd2-F	GAGTGGGAACTGGTAGTGTTG
Ccnd2-R	CGCACAGAGCGATGAAGGT
Cdc20-F	TTGGCCATGGTTGGATACTT
Cdc20-R	GAGTGCTGTGGATGTGCATT
Ccna2-F	GTGGTGATTCAAAACTGCCA
Ccna2-R	GGCCAGCTGAGCTTAAAGAA
Bcl2l1-F	CTGCATTGTTCCCGTAGAGA
Bcl2l1-R	GGTGAGTCGGATTGCAAGTT
GAPDH-F	CTAAGGCCAACCGTGAAA
GAPDH-R	AGAAGGAAGGCTGGAAAA
