# Supplementary material for: Iron-dependent histone 3 lysine 9 demethylation controls B cell proliferation and humoral immune responses
Source: Nat Commun. 2019 Jul 3;10:2935. doi: 10.1038/s41467-019-11002-5 (PMC6610088; doi:10.1038/s41467-019-11002-5)
Supplement: Supplementary file 3 — Reporting Summary [file 41467_2019_11002_MOESM3_ESM.pdf]

nature research

Corresponding author(s): Xiaoren Zhang; Fudi Wang

Last updated by author(s): May 26, 2019

# Reporting Summary

Nature Research wishes to improve the reproducibility of the work that we publish. This form provides structure for consistency and transparency in reporting. For further information on Nature Research policies, see Authors & Referees and the Editorial Policy Checklist.

## Statistics

For all statistical analyses, confirm that the following items are present in the figure legend, table legend, main text, or Methods section.

| n/a | Confirmed | |
|---|---|---|
| ☐ | ☒ | The exact sample size (*n*) for each experimental group/condition, given as a discrete number and unit of measurement |
| ☐ | ☒ | A statement on whether measurements were taken from distinct samples or whether the same sample was measured repeatedly |
| ☐ | ☒ | The statistical test(s) used AND whether they are one- or two-sided<br>*Only common tests should be described solely by name; describe more complex techniques in the Methods section.* |
| ☐ | ☒ | A description of all covariates tested |
| ☐ | ☒ | A description of any assumptions or corrections, such as tests of normality and adjustment for multiple comparisons |
| ☐ | ☒ | A full description of the statistical parameters including central tendency (e.g. means) or other basic estimates (e.g. regression coefficient) AND variation (e.g. standard deviation) or associated estimates of uncertainty (e.g. confidence intervals) |
| ☐ | ☒ | For null hypothesis testing, the test statistic (e.g. *F*, *t*, *r*) with confidence intervals, effect sizes, degrees of freedom and *P* value noted<br>*Give P values as exact values whenever suitable.* |
| ☒ | ☐ | For Bayesian analysis, information on the choice of priors and Markov chain Monte Carlo settings |
| ☒ | ☐ | For hierarchical and complex designs, identification of the appropriate level for tests and full reporting of outcomes |
| ☒ | ☐ | Estimates of effect sizes (e.g. Cohen's *d*, Pearson's *r*), indicating how they were calculated |

*Our web collection on statistics for biologists contains articles on many of the points above.*

## Software and code

Policy information about availability of computer code

| | |
|---|---|
| Data collection | A detailed description of the software and code has been included in the Methods section. |
| Data analysis | A detailed description of the software and code has been included in the Methods section. |

For manuscripts utilizing custom algorithms or software that are central to the research but not yet described in published literature, software must be made available to editors/reviewers. We strongly encourage code deposition in a community repository (e.g. GitHub). See the Nature Research guidelines for submitting code & software for further information.

## Data

Policy information about availability of data

All manuscripts must include a data availability statement. This statement should provide the following information, where applicable:

- Accession codes, unique identifiers, or web links for publicly available datasets
- A list of figures that have associated raw data
- A description of any restrictions on data availability

The RNA-seq and ChIP-seq data that support the findings of this study have been deposited in Gene Expression Omnibus (NCBI) and are accessible through GEO Series accession numbers GSE100455 and GSE120073.
The following figures have associated raw data: Fig. 6a; Fig. 7c and d.

# Field-specific reporting

Please select the one below that is the best fit for your research. If you are not sure, read the appropriate sections before making your selection.

☒ Life sciences ☐ Behavioural & social sciences ☐ Ecological, evolutionary & environmental sciences

For a reference copy of the document with all sections, see [nature.com/documents/nr-reporting-summary-flat.pdf](nature.com/documents/nr-reporting-summary-flat.pdf)

# Life sciences study design

All studies must disclose on these points even when the disclosure is negative.

| | |
|---|---|
| Sample size | No sample size estimation was performed for experiments involving primary B cells, mouse experiments and population analysis. Sample size was based on the magnitude of effect observed, whether or not statistical significance was reached. |
| Data exclusions | No data was excluded from the analysis. |
| Replication | For all experiments, at least two independent biological replicates were performed, and data were representative of two independent experiments. The number of replicates is presented in each figure legend. |
| Randomization | Primary B cell isolated from wildtype C57BL/6 mice were randomly allocated to groups before different treatments. |
| Blinding | Blinding was not performed. It was not considered necessary because all the measurements were done using objective methods. |

# Reporting for specific materials, systems and methods

We require information from authors about some types of materials, experimental systems and methods used in many studies. Here, indicate whether each material, system or method listed is relevant to your study. If you are not sure if a list item applies to your research, read the appropriate section before selecting a response.

## Materials & experimental systems

| n/a | Involved in the study | |
|---|---|---|
| ☐ | ☒ | Antibodies |
| ☒ | ☐ | Eukaryotic cell lines |
| ☒ | ☐ | Palaeontology |
| ☐ | ☒ | Animals and other organisms |
| ☐ | ☒ | Human research participants |
| ☒ | ☐ | Clinical data |

## Methods

| n/a | Involved in the study | |
|---|---|---|
| ☐ | ☒ | ChIP-seq |
| ☐ | ☒ | Flow cytometry |
| ☒ | ☐ | MRI-based neuroimaging |

## Antibodies

| | |
|---|---|
| Antibodies used | For western blot<br>Anti-phospho ERK1/2 (9101), anti-ERK1/2 (9102), anti-phospho RelA (3033), anti-phospho p38 (9211), and anti-p38 (9212) antibodies were purchased from Cell Signaling Technology. An anti-cyclin E1 antibody (11554-1-AP) was purchased from Proteintech. An anti-GAPDH (KC-5G5) antibody was purchased from Kangchen. An anti-actin antibody (A5441) was purchased from Sigma-Aldrich.<br>For immunofluorescence<br>PerCP-conjugated anti-B220 (553093), FITC-conjugated anti-GL7(562080), PE-conjugated anti-Fas (554258), APC-conjugated anti-IgD (560868) antibodies, APC-conjugated anti-CD21(558658), FITC-conjugated anti-CD23(553138) and PE-conjugated anti-IgM(553409) were purchased from BD Biosciences. Alexa Fluor 488-conjugated rat anti-IgD (562023, BD Pharmingen), biotin-conjugated peanut agglutinin (PNA, BA-0074, Vector Laboratories), and Alexa Fluor 555-conjugated streptavidin (S32355, Invitrogen).<br>For ChIP<br>The antibodies for ChIP, anti-H3K9me2 (39239), anti-H3K9me3 (39161) were purchased from Active Motif. |
| Validation | Antibodies used in this study are commercially available,the specificity had been tested by the supplier. |

## Animals and other organisms

Policy information about [studies involving animals](); [ARRIVE guidelines]() recommended for reporting animal research

| | |
|---|---|
| Laboratory animals | Mouse lines were all in the C57BL6/J background. |

Wild animals: N/A

Field-collected samples: N/A

Ethics oversight: All animal experiments were performed in compliance with the NIH Guide for the Care and Use of Laboratory Animals (National Academies Press, 2011) and were approved by the Institutional Biomedical Research Ethics Committee of the Shanghai Institutes for Biological Sciences, Chinese Academy of Sciences.

Note that full information on the approval of the study protocol must also be provided in the manuscript.

# Human research participants

Policy information about studies involving human research participants

Population characteristics: The human population assessed in this study were selected from a previous seroprevalence survey of measles in Zhejiang Province, China. The survey consisted of a multistage design and was employed to obtain samples from 5 counties in Zhejiang Province after consideration of geographical and economic status.Measles vaccine is routine immunization of the population. In recent decades, Zhejiang Province routinely use two needles of measles vaccination for children aged 8 months and 18 months, with a dose of 0.5 ml. The source of the vaccine is China Biotechnology Co., Ltd.

Recruitment: Serum samples from individuals who were known to be affected by an acute infection were excluded.

Ethics oversight: The study was approved by the ethics committee of Zhejiang Provincial Center for Disease Control and Prevention, and complied with the principles expressed at the Declaration at Helsinki. Written informed consent was obtained from all participants or their guardians (for children younger than 18 years old) before enrollment in the study.

Note that full information on the approval of the study protocol must also be provided in the manuscript.

# ChIP-seq

## Data deposition

- [x] Confirm that both raw and final processed data have been deposited in a public database such as GEO.
- [x] Confirm that you have deposited or provided access to graph files (e.g. BED files) for the called peaks.

Data access links
*May remain private before publication.*: https://www.ncbi.nlm.nih.gov/geo/query/acc.cgi?acc=GSE131716

Files in database submission:
S1.wig (control B cells)
S2.wig (control B cells with anti-IgM stimulation)
S3.wig (iron-deficient B cells with anti-IgM stimulation)
s1.fastq (control B cells)
S2.fastq (control B cells with anti-IgM stimulation)
S3.fastq (iron-deficient B cells with anti-IgM stimulation)

Genome browser session (e.g. UCSC): N/A

## Methodology

Replicates: N/A

Sequencing depth: Single-end, 50bp

Antibodies: Anti-H3K9me2 (Active motif, 39239)

Peak calling parameters: Whole genome Peaks scan refer to Model-based Analysis of ChIP-Seq (Genome Biology 2008, 9:R137), for the predicted Peaks, the Poisson distribution model is used to calculate the p-value of each potential region using the aligned Reads. When p-value < 1e-5, this area is considered to be a Peaks.

Data quality: Peaks were considered with p < 1e-5.

Software: MACS version 2.1.0

## Flow Cytometry

### Plots

Confirm that:

☒ The axis labels state the marker and fluorochrome used (e.g. CD4-FITC).

☒ The axis scales are clearly visible. Include numbers along axes only for bottom left plot of group (a 'group' is an analysis of identical markers).

☒ All plots are contour plots with outliers or pseudocolor plots.

☒ A numerical value for number of cells or percentage (with statistics) is provided.

### Methodology

| | |
|---|---|
| Sample preparation | Details provided in the methods section. |
| Instrument | FACS Calibur |
| Software | FlowJo V 10 |
| Cell population abundance | Purity was in excess of 95% for splenic primary B cell subset analysed post MACS separation. |
| Gating strategy | Gating strategies are present in the supplementary information. |

☒ Tick this box to confirm that a figure exemplifying the gating strategy is provided in the Supplementary Information.
